# Targeting Natural Plant Metabolites for Hunting SARS-CoV-2 Omicron BA.1 Variant Inhibitors: Extraction, Molecular Docking, Molecular Dynamics, and Physicochemical Properties Study

**DOI:** 10.3390/cimb44100342

**Published:** 2022-10-19

**Authors:** Heba Ali Hassan, Ahmed R. Hassan, Eslam A. R. Mohamed, Ahmad Al-Khdhairawi, Hala E. Taha, Hanan M. El-Tantawy, Iman A. M. Abdel-Rahman, Ali E. Raslan, Khaled S. Allemailem, Ahmad Almatroudi, Faris Alrumaihi, Maha A. Alshiekheid, Hafiz Muzzammel Rehman, Mahmoud M. Abdelhamid, Islam M. Abdel-Rahman, Ahmed E. Allam

**Affiliations:** 1 Department of Pharmacognosy, Faculty of Pharmacy, Sohag University, Sohag 82524, Egypt; 2 Medicinal and Aromatic Plants Department, Desert Research Center, El-Matariya, Cairo 11753, Egypt; 3 Department of Chemistry, Faculty of Science, Minia University, Minia 61511, Egypt; 4 Department of Biological Science and Biotechnology, Faculty of Science and Technology, Universiti Kebangsaan Malaysia, Bangi 43600, Selangor, Malaysia; 5 Department of Pharmacognosy, Faculty of Pharmacy, South Valley University, Qena 83523, Egypt; 6 Department of Pharmacognosy, Faculty of Pharmacy, Al-Azhar University, Assiut 71524, Egypt; 7 Department of Medical Laboratories, College of Applied Medical Sciences, Qassim University, Buraydah 51452, Saudi Arabia; 8 Department of Botany & Microbiology, College of Science, King Saud University, Riyadh 11451, Saudi Arabia; 9 Department of Human Genetics and Molecular Biology, University of Health Sciences, Punjab 54600, Pakistan; 10 Department of Pharmaceutical Chemistry, Faculty of Pharmacy, Al-Azhar University, Assiut 71524, Egypt; 11 Department of Pharmaceutical Chemistry, Faculty of Pharmacy, Deraya University, New-Minia 61519, Egypt

**Keywords:** *Echium angustifolium*, peach fruits, SARS-COV-2 Omicron, molecular docking, molecular dynamics

## Abstract

(1) Background: SARS-CoV-2 Omicron BA.1 is the most common variation found in most countries and is responsible for 99% of cases in the United States. To overcome this challenge, there is an urgent need to discover effective inhibitors to prevent the emerging BA.1 variant. Natural products, particularly flavonoids, have had widespread success in reducing COVID-19 prevalence. (2) Methods: In the ongoing study, fifteen compounds were annotated from Echium angustifolium and peach (Prunus persica), which were computationally analyzed using various in silico techniques. Molecular docking calculations were performed for the identified phytochemicals to investigate their efficacy. Molecular dynamics (MD) simulations over 200 ns followed by molecular mechanics Poisson–Boltzmann surface area calculations (MM/PBSA) were performed to estimate the binding energy. Bioactivity was also calculated for the best components in terms of drug likeness and drug score. (3) Results: The data obtained from the molecular docking study demonstrated that five compounds exhibited remarkable potency, with docking scores greater than −9.0 kcal/mol. Among them, compounds **1, 2** and **4** showed higher stability within the active site of Omicron BA.1, with ΔG_binding_ values of −49.02, −48.07, and −67.47 KJ/mol, respectively. These findings imply that the discovered phytoconstituents are promising in the search for anti-Omicron BA.1 drugs and should be investigated in future in vitro and in vivo research.

## 1. Introduction

The World Health Organization (WHO) was informed on 31 December 2019 of a cluster of cases of pneumonia of unknown etiology first detected in Wuhan city, Hubei province, China [1]. A new coronavirus (SARS-CoV-2) with high transmissibility was discovered, and this unusual disease, known as Coronavirus Disease 2019 (COVID-19), spread rapidly across the world [2]. The WHO reported approximately 440 million SARS-CoV-2 infections and over 6 million confirmed deaths worldwide by 9 March 2022. The WHO has tracked and assessed the evolution of SARS-CoV-2 since its emergence and identified certain Variants of Concern (VOCs) that pose an increased risk worldwide. To date, numerous SARS-CoV-2 VOCs have been found with groups of mutations in the spike protein or other regions in the viral genome that have been associated with increased disease spread or immune evasion [3].

The Omicron variation indicates the beginning of a new phase of the COVID-19 pandemic [4,5,6,7]. In November 2021, a new SARS-CoV-2 strain (Omicron) with higher transmissibility was detected for the first time in South Africa [8]. Unlike previous SARS-CoV-2 strains, which mainly infect the lungs and cause severe disease, the Omicron variant mainly infects the respiratory tract and is characterized by milder symptoms [9,10]. The receptor-binding domain (RBD) of SARS-CoV-2 binds to human angiotensin-converting enzyme 2 (ACE2), which is found on the surface cells of the pharynx and the epithelial cells of the lung [11]. This binding induces a fusion between the human S protein and the human cell membrane, resulting in a duplication of genetic material with the host cells [12]. Omicron BA.1 has thirty-seven amino acid mutations in the spike (S) protein of the virus, fifteen of which are in the RBD [13,14]. The large number of mutations, especially that of N501Y, in the RBD of Omicron BA.1 resulted in higher ACE2 binding than the prototypical RBD of the earliest Wuhan strain [15].

In detail, the spike protein mediates SARS-CoV-2 penetration into host cells via its binding to host receptors. It consists of the S1 and S2 subunits [16]. The RBD, which binds to ACE2, is found in the S1 subunit, whereas the transmembrane region is found in the S2 subunit. The transmembrane region helps in attachment of the spike protein to the membrane and enables the fusion of both cellular and viral membranes [17,18]. A crucial phase in the infection cycle is the cleavage of the spike protein at the S1–S2 and S2 sites with the help of furin [19] and type II transmembrane serine protease (TMPRSS2) [20] or cathepsin L [21]. Cleavage via TMPRSS2 and cathepsin L at the S2 site promotes two separate SARS-CoV-2 penetration pathways. Due to its location on the cell surface, TMPRSS2 facilitates the entrance through the plasma membrane, whereas cathepsin L is responsible for mediating the entrance through the endosome [17]. The Omicron variant has a comparable infection cycle but is more contagious as compared to earlier forms. Many investigations have revealed that Omicron RBD has a 1.5–2.8-fold greater binding affinity to ACE2 than the wild type [14,22].

Conventional drug research and development can take several years, and the cost of bringing a new drug to market can run into billions of dollars [23]. In lieu of all this effort and time, computer-aided drug design (CADD) can be a fast, efficient and cost-effective way to develop innovative drugs [24,25].

In addition, a number of previous studies highlighted the interaction of bioactive compounds (Cmds), especially plant flavonoids, with SARS-CoV-2 proteins to combat the COVID-19 pandemic [26,27,28], which encouraged us to conduct our research. Thus, a total of fifteen Cmds were identified from two plant sources Three of them (**1**,**13**, and **15**) were extracted and annotated from *Echium angustifolium* aerial parts (Boraginaceae family) via LC–ESI–MS/MS [29]. However, the other phytochemicals studied (**2–12** and **14**) were isolated and identified from peach (*Prunus persica* (L.) Batsch), one of the most nutritionally important fruits in the world. In terms of biological activity, peach fruits were reported to contain abundant phenolic acids, flavanones and chalcones, which exhibited a wide range of activities against viral infections [30,31,32]. At that time, Traditional Chinese Medicine (TCM) recommended peach as a contributor to the prevention and treatment of COVID-19 [33], which prompted us to conduct this study. Several previous in vitro and in silico studies highlighted the importance of using compounds extracted from natural sources, in particular, those extracted from *Echium angustifolium* and peach in combating various diseases [34,35].

A molecular docking and molecular dynamics investigation was carried out in the current research to study the efficacy of the identified phytochemicals and to comprehend the nature of the interaction with omicron. In view of achieving a better understanding of the potency of the investigated inhibitors, a drug-likeness analysis was carried out to assess the bioeffects and bioavailability of the best Cmds identified from the molecular docking and dynamics study. The current findings will serve as the foundation for future in vivo and in vitro investigations to ensure the efficacy of the developed anti-Omicron BA.1 drugs.

## 2. Materials and Methods

### 2.1. Plant Material

The aerial parts of *Echium angustifolium* Mill. were collected from Mersa Matruh (Agiba region), Egypt in April 2020., as well as peach plant aerial parts were collected in March 2020 from Upper Egypt. The plant samples were authenticated by staff members of Plant Taxonomy, Faculty of Science, Assiut University, Egypt. Their voucher specimens (T-21 and V-21, respectively) were deposited.

### 2.2. Extraction and Isolation

Air-dried aerial parts of *E. angustifolium* were ground to yield 1 kg of plant powder. This powder was extracted at room temperature by maceration with 80% EtOH (3 × 4 L, every 48 h). The obtained extracts were combined and vacuum-concentrated at 45 °C, yielding 73 g of the total extract. This dried extract was then fractionated by suspending in 70% MeOH (400 mL) in a separating funnel and partitioning with *n*-hexane, yielding 50 g of defatted fraction [29]. This fraction was then fractionated on polyamide 6S column chromatography using the eluent of H_2_O-EtOH (100:0 → 0:100). All obtained fractions were screened using silica gel 60 GF254 NP-TLC plates (Merck, Darmstadt, Germany), yielding a promising sub-fraction. Furthermore, LC–ESI–MS/MS was used to identify the chemical constituents (compound **1**, **13**, and **15**) in the Ea-DfD fraction using Acquity UPLC™ (Waters, Milford, MA, USA) in both ionization modes [36].

For the other plant constituents’ extraction, air-dried aerial parts of peach fruits were extracted three times with EtOH (5 L of each) at room temperature to yield the ethanol extract (63 g). The ethanol extract was suspended in distilled water and divided between *n*-hexane, ethyl acetate and the remaining water to give the *n*-hexane fraction (12 g), ethyl acetate fraction (5.1 g) and the remaining aqueous fraction (45.5 g). The ethyl acetate fraction from the ethanol extract was sub-fractionated on a silica gel column using chloroform-methanol gradient elution (25%, 50%, 75% and 100%; 2 L each). The fraction eluted by 50% methanol (2.3 g) was further separated by chromatography on an ODS column (80 × 200 mm; Cosmosil 140 C-18 PREP, Nacalai Tesque, Tokyo, Japan) using six mobile phase systems of methanol-water (10%, 25%, 40%, 50%, 70% and 90% *v/v*; elution volume: 1.5 L of each) to give six corresponding fractions. The fraction eluted with 25% methanol was further chromatographed by column chromatography on silica gel and eluted by a stepwise gradient of chloroform-methanol (ratios of 9:1, 85:15, 8:2, 7:3 and 6:4; *v/v* elution volume: 200 mL each) to give five corresponding fractions. The fraction eluted with 85:15 of chloroform-methanol resulted in elution of **2–6** (23, 38, 33, 26 and 38 mg, respectively). The fraction eluted with 8:2 resulted in elution of **7–10** (28, 35, 34, and 38 mg, respectively). Additionally, that eluted with 7:3 of chloroform-methanol resulted in elution of **11, 12,** and **14** (22, 29, and 34 mg, respectively).

### 2.3. Protein Preparation

All in silico calculations were performed using the three-dimensional crystal structure of the SARS-CoV-2 Omicron Variant BA.1 receptor-binding domain (RBD) (PDB ID: 7U0N, resolution: 2.61 Å). The crystal structure of the wild-type SARS-CoV-2 RBD was also investigated (PDB ID: 6M0J, resolution: 2.45 Å). Ions, water molecules, and hetero-atoms were removed from the downloaded viral protein. The H++ web service (http://biophysics.cs.vt.edu/H++, last accessed on 14 May 2022)was employed to study the protonation states of protein amino acids [37,38]. As well, all of the missing hydrogen atoms were added. Physiological parameters such as pH, salinity, internal dielectric, and external dielectric were set to 7.0, 0.15, 10, and 80, respectively, for the H++ computations.

### 2.4. Inhibitor Preparation

Chem3D Pro 12.0 software (version 12.0.2, Cambridge, MA, USA) was exploited in order to sketch and analyze the chemical structures of the fifteen extracted Cmds. The MM2 force field was used to minimize energy in all of the Cmds tested. Such an energy minimization procedure is required prior to molecular docking research to limit the influence of any potential unfavorable torsion angles, bond angles, bond lengths, or undesired non-bonded interactions [39].

### 2.5. Molecular Docking

Molecular docking is widely regarded as the most effective approach in computational drug development for determining the efficacy of drugs under investigation [40]. The binding affinities of these Cmds were investigated using AutoDock Vina (California, CA, USA) [41]. In this investigation, all parameters were left at their normal settings, with the exception of the exhaustiveness parameter, which was set to 200. The BA.1-RBD residues were encased in a docking grid box with XYZ dimensions of 25 × 25 × 25 (Å). Furthermore, the grid spacing was adjusted to 1.0 Å. The resulting nine docked inhibitor poses were examined, and the best one was chosen. The protein–ligand interactions were visualized using BIOVIA Discovery Studio (Waltham, MA, USA) [42]. Table 1 illustrates the mutated residues in BA.1 and the original residues of the wild-type SARS-CoV-2.

### 2.6. Molecular Dynamics Simulations

The YASARA Structure (version 22.5.22, YASARA Biosciences GmbH, Vienna, Austria) protocol was used for molecular dynamics (MD) simulations [43] The main objective of employing MD simulation is to generate deep insight into the protein–ligand complexes’ stability. The AMBER14 force field (San Francisco, CA, USA) was the force field used in the MD simulations. Firstly, hydrogen atoms, atomic partial charges, and force field parameters were well studied as the primary structure preparation step. Following system preparation, 5000 step energy minimizations were performed using a combination of steepest and conjugate gradient algorithms to clear any inappropriate geometries or steric clashes. The MD simulations were performed on amino acid residues at the default physiological pH setting (7.4). Water molecules were successfully presented into the system at constant temperature and pressure. To maintain the systems’ neutral state, counter ions (Na+ or Cl) at a concentration of 0.9% were added. The Berendsen barostat technique was employed to keep the pressure at 1 atm [44]. The particle-mesh Ewald (PME) method was used to calculate the long-range coulomb forces [45,46]. For non-bonded interactions, the cut-off radius was set to 8 Å. The Langevin thermostat method [47] was used to keep the temperature at 298.15 K through the heating step of the docked protein–ligand complexes from 0 K to 300 K over 10 ps. The periodic boundary conditions were also considered. In each case, the cubic simulation cell was designed to be 20 Å larger than the examined protein–ligand complexes. A consistent simulation speed was maintained for intramolecular processes with a multiple time step of 1.25 fs. The SHAKE method was used to constrain all intermolecular bonds, including hydrogen bonds, at a 2 fs integration step [48]. As a pre-final step, the systems were sufficiently equilibrated for 1 ns. Finally, production processes were completed across simulation periods of 50, 100, 150, and 200 ns. Snapshots of the simulation trajectory were taken every 100 ps, as defined by the root-mean-square deviations (RMSDs) of the solutes from the original structure. The simulation steps were carried out with the help of a pre-installed macro (md_run.mcr) from the YASARA package. The Poisson–Boltzmann method is used in the YASARA program [49]. The surfcost parameter was set at 0.35 in order to calculate the entropic cost of exposing a Å2 to the solvent. The inhibitor’s binding energy was calculated using the AMBER14 force field. The binding energies calculated from PBS were divided by a factor of 20 to maintain compatibility with the empirically determined values [50]. Various types of post-MD analyses, such as the root-mean-square deviation (RMSD), radius of gyration (Rg), root-mean-square fluctuation (RMSF), and solvent-accessible surface area (SASA), were all used to study the behavior of the best identified Cmds over the 200 ns MD simulations.

### 2.7. Binding Energy Calculations

The molecular mechanics Poisson–Boltzmann surface area (MM/PBSA) technique was used to calculate the binding free energies of the examined Cmds against BA.1 [51]. The equations shown below were used to calculate the MM/PBSA binding free energy.

$$\Delta G_{binding}=\;\Delta G_{C}-\Delta G_{P}-\;\Delta G_{L}$$

$$\Delta G_{binding}=\Delta H-T\Delta S=\Delta E_{MM}+\Delta G_{Sol}-T\Delta S$$

The complex, protein, and ligand binding energies are described by Δ*G_C_*, Δ*G_P_*, and Δ*G_L_*, respectively. Furthermore, Δ*G_Sol_*, Δ*E_MM_* and −*TΔS* stand for solvation Gibbs energy, gas-phase molecular mechanics change, and conformational entropy, respectively. The term Δ*E_MM_* can be calculated by adding the van der Waals and electrostatic interactions. The term Δ*G_Sol_* is easily defined as the sum of the polar and non-polar solvation values. The term −*T∆S* denotes the entropic contribution

### 2.8. Assessment of Drug-Relevant Properties

For the sake of determining physicochemical properties, the Osiris property explorer (https://www.organic-chemistry.org/prog/peo/, last accessed on 21 May 2022) was used. To characterize the extent to which the discovered Cmds were toxic, tumorigenic, mutagenic, irritating, and reproductive qualities were expected. Partition coefficient between n-octanol and water (clogP), water solubility (logS), molecular weight (Mwt), and topological polar surface area (TPSA) were also investigated. All the above parameters were combined to produce a single value known as the drug score (DS), representing the total drug potential. DS can be estimated using the following equation:

$$DS=\prod \left(\frac{1}{2}+\frac{1}{2}S_{i}\right)\cdot \;\prod t_{i}$$

where (*S_i_*) is:

$$S_{i}=\frac{1}{1+e^{ap_{i}+b}}$$

*S_i_* represents the contributions estimated directly from cLogP (coctanol/cwater), molecular weight (Mwt), and drug-likeness (pi) via the second equation, which expresses a spline curve. Parameters a and b are (1, −5) for cLogP, (1, 5) for logS, (0.012, −6) for Mwt, and (1, 0) for drug-likeness. The ti parameter represents the contributions taken from the four major toxicity risk types (mutagenic, tumorigenic, irritant, and reproductive). The ti values are 1.0, 0.8, and 0.6 for no risk, medium risk, and high risk, respectively.

## 3. Results and Discussion

### 3.1. Identification of Phytoconstituents

Three active components; **1**, **13**, and **15** were identified from the aerial parts of *E. angustifolium*. Compound (**1**) was tentatively identified as echiumin A by comparing its LC–ESI–MS/MS results in the negative ion mode with the previous data [52]. According to our findings, Echiumin A (**1**) detected a molecular ion peak at *m/z* 1013.5 [M − H]^−^. However, MS data of compounds; **13** and **15** indicated that these two compounds could be identified as phenolic acid spermidine derivatives. Compound **15** produced molecular ions at *m/z* 468.3 [M + H]^+^, 466.2 [M − H]^−^ and fragments in positive ionization mode at 436.3 [M + H − 31]^+^, 292.2 [M + H − 176]^+^, and 147 [M + H − 321]^+^, assisted the occurrence of feruloyl and coumaroyl moieties bonded to spermidine moiety. By comparing our results with the published data [53], we annotated this compound as coumaroyl-feruloyl spermidine. Compound **13** was identified as diferuloyl spermidine that gave [M + H]^+^ at *m/z* 498.3, [M – H]^−^ at 496.2, and fragmentation patterns at *m/z* 463.2, 322.3 [M + H – 176]^+^, 177.1 [M + H – 321]^+^, and 353.3 [M – H – 143]^−^, consistent with the attachment of two feruloyl moieties with spermidine [53].

Besides; Twelve active components; **2–12** and **14** were identified from the aerial parts of peach based on literature data; kaempferol 3−O−(3”−O−*α*−rhamnopyranosyl)−α−rhamnopyranoside, compound **(2)** [54], Kaempferol3−O−[*α*−rhamnopyranosyl−(1→4)−O−α−rhamnopyranosyl−(1→6)−O]−β−galactopyranoside (kaempferol 3−O−*β*−isorhamninoside), compound **(3),** [55], kaempferol 3−O−*α*−rhamnopyranosyl−(1→6)−*β*− glucopyranosyl− 5−O−*α*−rhamnopyranoside, compound **(4)** [56], kaempferol 3−O−*α*−rhamnopyranosyl−(1→6)−*β*− glucopyranosyl− 7−O−*α*−rhamnopyranoside, compound **(5)** [55], kaempferol 3−O−*α*−rhamnopyranosyl (1→6)−*β*−galactopyranoside−7−O−α−rhamnopyranoside, compound **(6)** [57], kaempferol 3−O−α¬[(6P−coumaroyl galactopyranosyl−O−*β*−(1→4)−O−*α*−rhamnopyranosyl−(1→4)]−O−*α*−rhamnopyranoside, compound **(7)** [58], kaempferol 7−O−*α*−rhamnopyranoside, compound **(8)** [54], Kaempferol 3−O−*α*−arabinopyranosyl−5− O−*α*−rhamnopyranoside, compound **(9)** [59], Kaempferol 3−O− rutinoside, compound **(10)** [60], kaempferol −3−O−*α*−galactopyranosyl 7−O−α−rhamnopyranoside, compound **(11)** [56], kaempferol −3−O−*α*−rhamnopyranosyl (1→6) (3”−acetyl)−*β*−galactopyranoside, compound **(12)** [61], and kaempferol 3−O−*α*−arabinopyranosyl−7−O−*α*−rhamnopyranoside, compound **(14)** [56].

### 3.2. Molecular Docking

A total of fifteen plant substances were subjected to molecular docking calculations using AutoDock Vina to find out the best active Cmds. The names of the identified Cmds and their docking scores are listed in Tables S1 and S2 and Figure S1. The docking data showed that five Cmds had a docking score of ≥−9.0 kcal/mol kcal/mol against Omicron BA.1. Ligand binding is the fundamental step in the inhibition of protein activity. Accordingly, a comprehensive understanding of the interactions between small Cmds and proteins can serve as the basis for a rational method for drug development.

In Table 2, the docking scores, 2D chemical structures, and binding characteristics for the five most powerful phytochemicals with Omicron BA.1 are all summarized.

**Figure 1:**
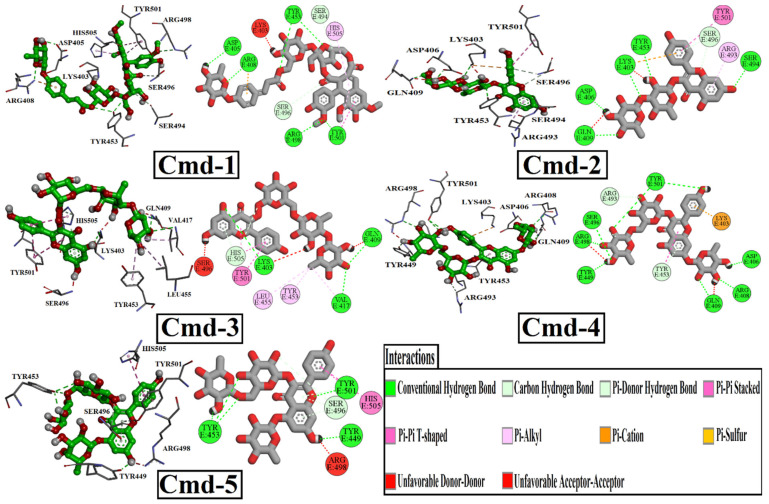
The 2D and 3D representations of the anticipated binding poses of the best-investigated phytochemical Cmds inside the active site of Omicron BA.1.

In addition, Figure 1 depicts the 2D representations of interactions of those five powerful Cmds with the crucial amino acid residues of Omicron BA.1. According to the docking features (Table 2) and binding modes (Figure 1), all discovered Cmds displayed similar binding behaviors, with an influential hydrogen bond with SER496, ARG498, and TYR501, and additional hydrogen bonds with multiple residues inside the Omicron BA.1 binding pocket.

In detail, Cmd-1 (docking score = −9.7 kcal/mol) formed a carbon-hydrogen bond with SER494 (3.35 Å), and SER 496 (3.75 Å). Interaction of pi-pi stacked type with TYR501 (4.74 Å). Interaction of pi-alkyl with TYR501 (4.45 Å) and HIS505 (4.57 Å). Cmd-1 formed six conventional hydrogen bonds with ASP405 (2.28 Å), ARG408 (2.80 Å), TYR453 (2.86, 3.13 Å), ARG498 (3.31 Å), and TYR501 (3.10 Å). Cmd-2, with a docking score of −9.5 kcal/mol, exhibited a featured pi-cation interaction with LYS403 (4.85 Å). It also formed pi-pi stacked and pi-alkyl interactions with TYR501 (5.50 Å) and ARG493 (4.58 Å), respectively. It also formed a carbon-hydrogen bond with SER496 (3.71 Å), a pi-donor hydrogen bond with the same residue at 4.02 Å, and several conventional hydrogen bonds with the following residues: LYS403 (2.99 Å), ASP406 (2.02 Å), GLN409 (3.08 Å), TYR453 (3.06 Å), SER494 (3.22 Å). Cmd-3, with a docking score of −9.2 kcal/mol, interacted with TYR501 in two ways: pi-pi stacked (4.41 Å) and pi-pi T-shaped (4.84 Å). It also interacted with HIS505 in a pi-pi T-shape (4.21 Å). Pi-alkyl interaction was observed with three different amino acid residues, VAL417 (4.29 Å), TYR453 (4.83 Å), and LEU455 (5.10 Å). It formed three main conventional hydrogen bonds with the following residues: LYS403 (3.22 Å), GLN409 (2.83 Å), and VAL417 (3.27 Å). Cmd-4 and Cmd-5 obtained the same docking score (−9.0 kcal/mol). However, they interacted with different residues inside the binding pocket. As in Cmd-2, Cmd-4 also showed pi-cation interaction with LYS403, but at different distances. Cmd-4 recorded one pi-pi T-shaped interaction with TYR453 (5.62 Å) and one pi-donor hydrogen bond with the same residue at 3.98 Å. One Carbon-hydrogen bond was detected with ARG493 at 3.67 Å. Cmd-4 exhibited eight conventional hydrogen bonds with ASP406 (2.13 Å), ARG408 (2.92 Å), GLN409 (2.90 Å), TYR449 (2.45 Å), SER496 (3.01 Å), ARG498 (3.09 Å), and TYR501 (2.75, 3.13 Å). Regarding Cmd-5, TYR453 and SER496 were detected as the main residues of interaction as they formed three conventional hydrogen bonds and three carbon-hydrogen bonds, respectively. One pi-donor hydrogen bond was noticed with TYR501, one pi-pi stacked interaction with the same residue, and one pi-pi-T-shaped interaction with HIS505 was also detected.

**Figure 2:**
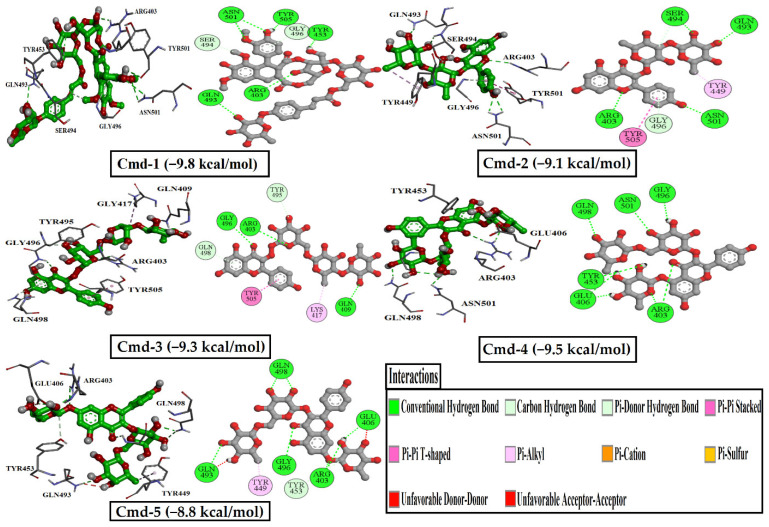
Depiction of binding modes and docking score for the best-investigated phytochemical Cmds with the wild-type RBD of SARS-CoV-2.

For comparison, the best-ranked five compounds were also docked to the identified RBD residues of the wild-type SARS-CoV-2. Figure 2 shows the results of docking analyses in terms of binding mode and binding affinity (kcal/mol).

Details of interacting residues of wild-type RBD-ligand complexes were examined in order to investigate various interactions in docked complexes. With respect to Cmd-1, six conventional hydrogen bonds were formed with the following residues; ARG403 (2.30 Å), TYR453 (2.25 Å), GLN493 (2.84 Å), ASN501 (2.85, 3.05 Å), and TYR505 (2.53 Å). Cmd-1 also made a carbon-hydrogen bond with SER 494 (3.49 Å), as well as a pi-donor hydrogen bond with GLY496 (2.94 Å).

Contrary to Cmd-1, two additional types of interactions can be noticed, Pi-Alkyl interaction with TYR449 (4.60 Å) and Pi-Pi T-shaped interaction with TYR505 (4.92 Å). It also formed four conventional hydrogen bonds: ARG403 (2.92 Å), GLN493 (2.35 Å), SER494 (2.39 Å), one carbon-hydrogen bond with SER494 (3.65 Å), and one pi-donor hydrogen bond with GLY496 (3.26 Å).

As in Cmd-2 docked complex, Cmd-3 demonstrated similar interactions with the wild RBD.Cmd-4, the second-best docking score inhibitor, interacted with the wild RBD via only one form of interaction: conventional hydrogen bonding. hydrogen bonding residues are: ARG403 (2.35, 2.68 Å), GLU406 (2.42, 2.43 Å), TYR453 (2.11 Å), GLY496 (2.51 Å), GLN498 (2.38 Å), and ASN501 (2.94 Å). Cmd-5, the least wild RBD inhibitors, with a docking score ≥ −9.0 kcal/mol, exhibited two different types of unfavorable interactions, unfavorable Acceptor-Acceptor with GLU406 (2.91 Å), and unfavorable donner-donner GLN493 (2.60 Å). It formed a double conventional hydrogen bond with ARG403 (2.38, 2.85 Å) and GLN498 (2.27, 2.98 Å), one single hydrogen bond with GLU406 (2.11 Å), GLN493 (2.29 Å), and GLY496 (2.69 Å). Only one weak pi-alkyl interaction was also observed with TYR449 at 5.11 A distance.

### 3.3. Molecular Dynamics (MD) Simulations

**Figure 3:**
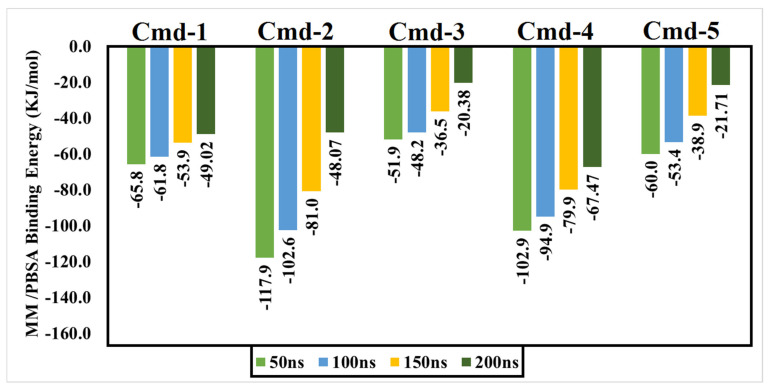
Estimated MM/PBSA binding energies for the best six drug candidates as Omicron BA.1 inhibitors.

Molecular dynamics (MD) simulations investigate the stability of receptor-inhibitor complexes, structural details, conformational elasticities and the reliability of receptor-inhibitor affinities. Accordingly, MD simulations over 200 ns followed by binding energy estimates for the top five components complexed with the Omicron BA. 1 were performed. The binding free energies were calculated using the MM/PBSA technique. The corresponding binding energies were estimated and shown in Figure 3.

Figure 3 shows that in the first 50 ns MD simulations, Cmd-2 and Cmd-4 had binding energies of more than −100.0 KJ/mol. Furthermore, values of binding energies were found to be over −60.0 KJ/mol for Cmd-1 and Cmd-5 and over −50.0 KJ/mol for Cmd-3. In order to increase the reliability of the obtained result, MD simulations were extended to 100 ns and the relevant binding energies were determined. What is noticeable about the estimated binding energies for five Cmds over 100 ns is that Cmd-1 and Cmd-2 still have binding energies of over −60.0 and −100.0 KJ/mol, respectively.

Moreover, computed binding energies over 100 ns for Cmd-3, Cmd-4, and Cmd-5 were slightly lowered by −3.7, −8.0, and −6.6 KJ/mol, respectively. Longer MD simulations of 150 ns and subsequently 200 ns were accomplished for those five potent Cmds in complex with Omicron BA.1 to obtain more reliable results, and the related binding affinities were calculated.

With respect to data from 200 ns MD simulation, Cmd-1 and Cmd-2 obtained binding energies of over −45.0 kJ/mol, whereas Cmd-4 had the highest stability compared to others, with a binding energy value of over −65.0 KJ/mol.

### 3.4. Post-MD Analyses

To verify the consistency and behavior of the discovered Cmds, their complexes with Omicron BA.1 were structurally and energetically analyzed over 200 ns MD simulations and compared to those of the apoprotein structure. The structural stability of the investigated complexes was assessed by measuring root-mean-square deviation (RMSD), root-mean-square fluctuation (RMSF), the radius of gyration (Rg), and solvent-accessible surface area (SASA).

#### 3.4.1. Root-Mean-Square Deviation (RMSD)

**Figure 4:**
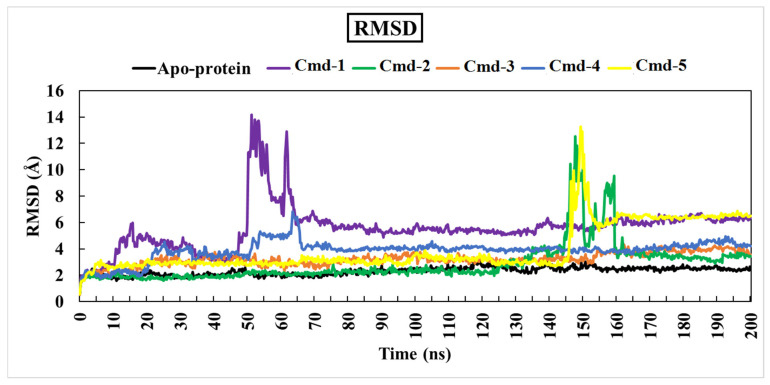
Root-mean-square-deviation (RMSD) of Omicron BA.1 backbone atoms from the initial structure complexed with the highest-ranked Cmds over 200 ns MD simulations.

**Figure 5:**
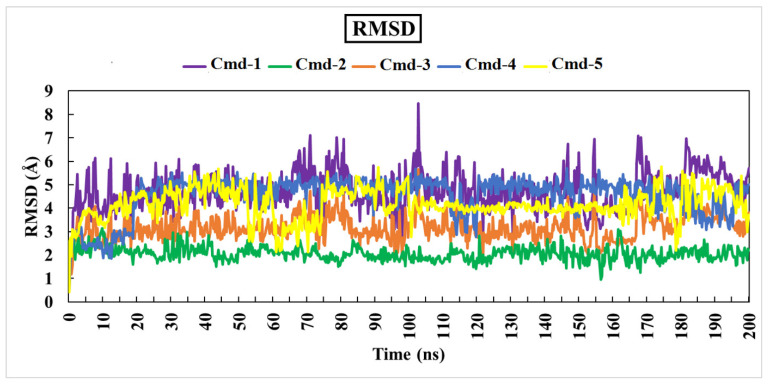
Variations in the RMSD values for the studied ligands over the course of the molecular dynamics simulation.

As previously mentioned, the primary goal of the MD simulations is to examine the positional and conformational changes of ligands when they bind to the binding pocket, which provides insight into the binding stability. In order to analyze the structural stability of the best five plant substances in a complex with Omicron BA.1, the root-mean-square deviation (RMSD) of the complete complex backbone atoms was evaluated for comparison purposes. RMSD values indicate the stability of the structures generated during the simulation trajectories [62,63]. RMSD values for the studied phytochemicals in the complex with Omicron BA.1 are plotted in Figure 4. Moreover, variations in the RMSD values for the studied ligands are also plotted in Figure 5.

Lower values of RMSD demonstrate an insignificant difference between the starting and final structures over the MD course.

**Figure 6:**
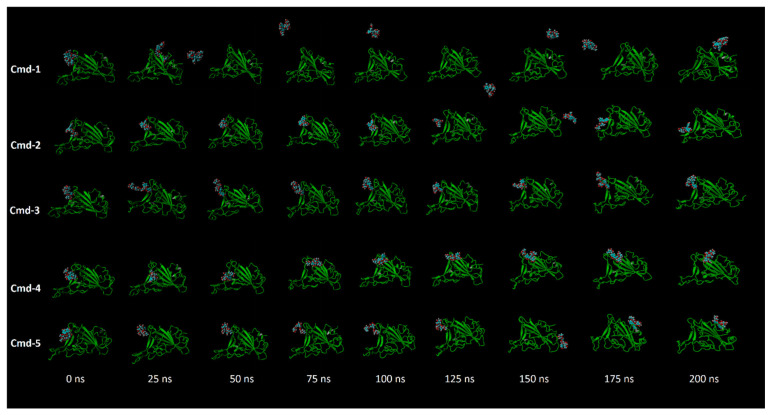
Snapshots for the movement of the studied five compounds inside the binding cavity of Omicron BA.1 during the MD simulation period.

Throughout the 200 ns MD simulations, the estimated RMSD values for Cmd-2 were found to be 2.81 Å with a difference of 0.51 Å from the apoprotein structure (2.30 Å). As well, Cmd-3, Cmd-4, and Cmd-5 obtained an average RMSD of 3.22, 3.87, and 3.96 Å, respectively. In contrast, Cmd-1 showed higher values of RMSD (avg. = 5.53 Å), which reflects the occurrence of some positional or conformational changes. As shown in Figure 6, the ligand movement for Cmd-2 and Cmd-3 within the binding pocket reflects the RMSD value, which is the lowest compared to the rest of the compounds.

#### 3.4.2. Root-Mean-Square Fluctuation (RMSF)

A root-mean-square fluctuation (RMSF) analysis was executed in order to study the flexibility of the Omicron BA.1 residues over the 200 ns MD simulations. This is a numerical measurement comparable to RMSD, except that instead of expressing positional changes across complete structures over time, RMSF calculates individual residue flexibility or how much a specific residue moves (fluctuates) throughout a simulation [64].

**Figure 7:**
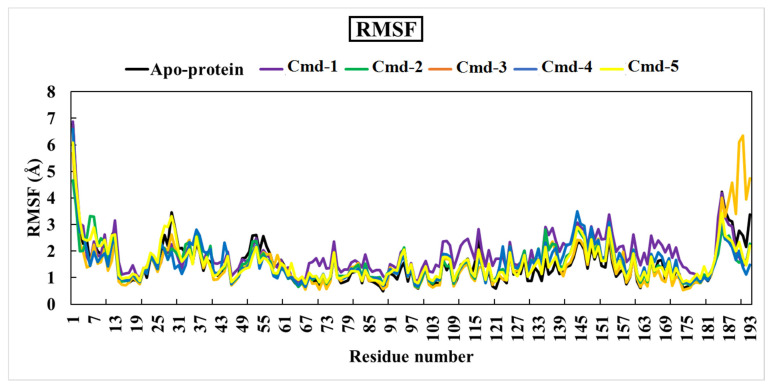
Root-mean-square-fluctuation (RMSF) of the apoprotein structure and five selected complexes (Omicron BA.1/Cmd-1, Omicron BA.1/Cmd-2, Omicron BA.1/Cmd-3, Omicron BA.1/Cmd-4, Omicron BA.1/Cmd-5).

RMSF per residue is often displayed vs. residue number and can indicate which amino acids in a protein contribute the most to molecular movement structurally. A high RMSF value indicates greater flexibility, whereas a low RMSF value indicates limited mobility. The RMSF graph for the five studied components and the apoprotein structure is illustrated in Figure 7.

The averages values of RMSF for apoprotein structure, Cmd-1, Cmd-2, Cmd-3, Cmd-4, and Cmd-5 were 1.50, 1.86, 1.53, 1.54, 1.55, and 1.57 (Å), respectively.

#### 3.4.3. The Radius of Gyration (Rg)

The radius of gyration (Rg) is a parameter that shows how the protein’s structural compactness changes during simulation [65]. An Rg analysis reflects the protein’s flexibility and stability [65]. Protein has the lowest Rg values and, as a result, the tightest packing and the most stability.

**Figure 8:**
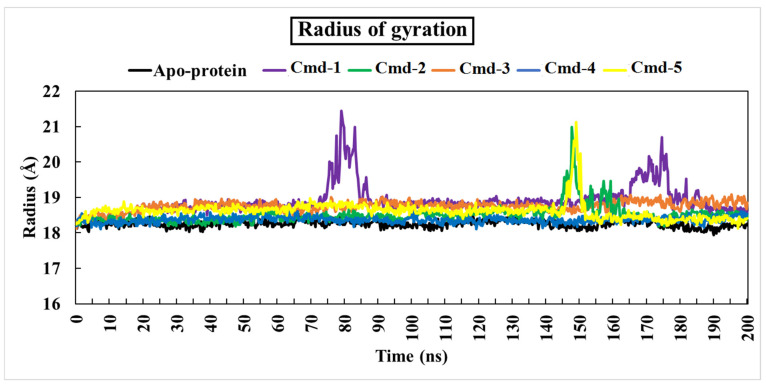
Radius of gyration (Rg) plot of apoprotein structure and five identified complexes through 200 ns MD simulations.

During the 200 ns simulation time, all Cmds demonstrated appropriate Rg behavior, as shown in Figure 8.

Compared to the apoprotein (Rg avg. = 18.25 Å), the Rg values for the five Cmds ranged from 18.35 to 18.88 (Å). Figure 5 further shows that Cmd-3 and Cmd-4 exhibited constant Rg behavior during the simulation, indicating that the protein structure is highly compact.

#### 3.4.4. The Solvent-Accessible Surface area Analysis (SASA)

**Figure 9:**
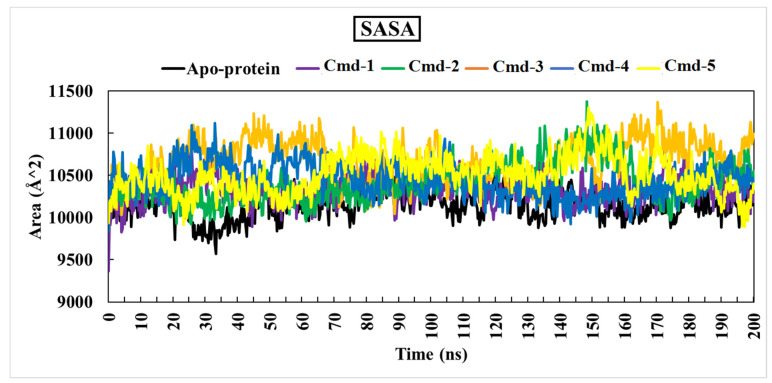
Solvent-accessible surface area (SASA) of apoprotein structure and the best five Cmds for 200 ns MD simulations.

Solvent-accessible surface area analysis (SASA) was computed to obtain a deeper insight into interactions between the complex and the solvent throughout the 200 ns MD simulation. Figure 9 depicts the SASA vs. simulation time curve for apoprotein and five complexes.

The average SASA values for Cmd-1, Cmd-2, Cmd-3, Cmd-4, and Cmd-5 were 10,317.33, 10,412.28, 10,669.97, 10,437.59 and 10,500.047 (Å^2^), respectively.

### 3.5. Drug-Relevant Properties

**Figure 10:**
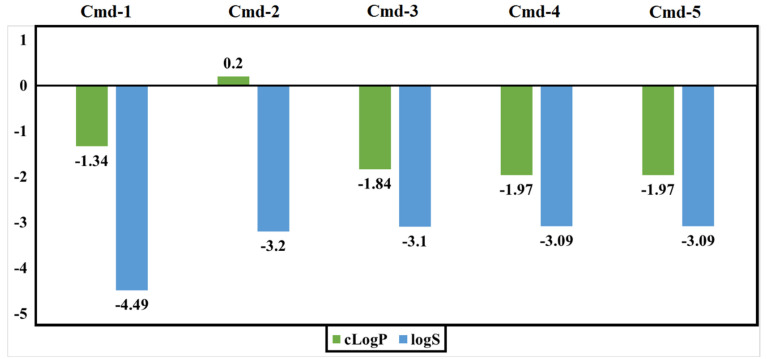
Predicted logS and cLogP values of the best identified Cmds.

Evaluation of molecular features should be carried out through the inspection of many parameters. For instance, drug solubility (logS) is a crucial element that influences drug transport from the site of administration into the bloodstream. It is also well recognized that low medication solubility can result in poor absorption. The logS value should be greater than −4.0. The second factor is the partition coefficient between n-octanol and water, cLogP, which is used to determine the molecule’s hydrophilicity. For the molecule to be well absorbed, values of cLogP should be in the range of −4.0 to 5.0. Figure 10 illustrates the estimated values of logS and cLogP for the five inspected Cmds. All Cmds are within the allowed range, implying good absorption.

**Figure 11:**
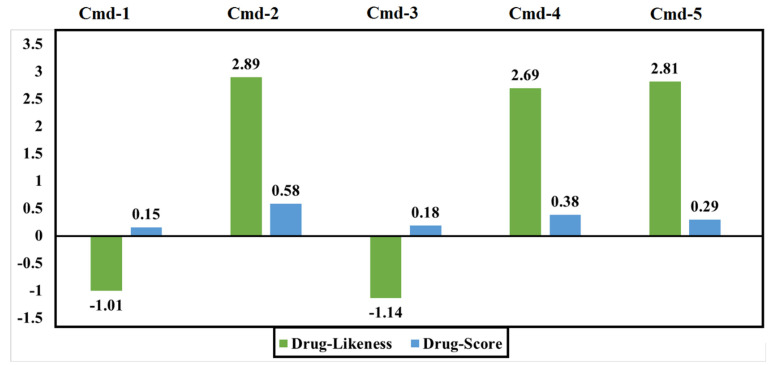
Calculated drug-likeness and drug scores of the best studied Cmds.

Numerical values of the drug-likeness parameter should be in the positive range. A positive value indicates that the molecule primarily comprises fragments that are commonly found in commercial medications. The drug score combines all computed values of drug-likeness, cLogP, logS, Mwt, and toxicity risks into a single useful value that can be used to assess the compound’s overall potential to qualify for drug approval. Figure 11 depicts the values of the estimated drug-likeness and drug score for the five phytoconstituents.

Both Cmd-1 and Cmd-3, which had negative drug-likeness values, had the lowest drug scores when compared to others. Strikingly, Cmd-2 obtained the highest values of drug-likeness and drug score when compared to other Cmds.

Another parameter to consider is toxicity, which indicates whether the molecule has a low risk (G), medium risk (Y), or high risk (R). The toxicity prediction is executed based on a precomputed collection of structural fragments that cause toxicity alerts if detected in the studied structure. Table 3 displays data from the toxicity evaluation for the five plant substances.

As shown in Table 3, Cmd-2 showed low toxicity risks towards the four inspected toxicity parameters. Cmd-1 and Cmd-3 exhibited high reproductive and tumorigenic toxicity risks, respectively. Excluding mutagenicity, Cmd-5 showed low tumorigenic, irritant, and reproductive toxicity risks. Cmd-4 has a medium reproductive side effect.

## 4. Conclusions

In this work, fifteen compounds from *Echium angustifolium* and peach were successfully identified and screened for possible inhibition of the SARS-CoV-2 Omicron BA.1 variant using various in silico methods. According to a molecular docking study, five phytochemicals showed promising inhibition of Omicron BA.1, with a docking score greater than −9.0 kcal/mol. The dynamic behavior of the five best docked protein–ligand complexes was studied on a time scale of 200 ns. This revealed different effects of the ligands on docking to Omicron BA.1, which were assessed by their respective RMSD, RMSF, Rg and SASA. Moreover, the analysis of molecular properties such as solubility (logS), hydrophilicity (cLogP), toxicity, drug-likeness, and drug score revealed that the studied components had a high chance of becoming drug-like compounds. The results of docking, dynamics and physicochemical property evaluation suggest that the annotated phytoconstituents from *Echium angustifolium and* peach are promising drug candidates. Further developments, especially in vitro and in vivo studies, are an important step to test these compounds as effective anti-Omicron BA.1 drugs.

## Figures and Tables

**Table 1:** Mutation sites in the RBD of Omicron BA.1 and the original residues of SARS-CoV-2.

Spike Protein	Mutation Sites														
	339	371	373	375	417	440	446	477	478	484	493	496	498	501	505
Wuhan-Hu-1 (wild type)	GLY	SER	SER	SER	LYS	ASN	GLY	SER	THR	GLU	GLN	GLY	GLN	ASN	TYR
Omicron BA.1 (mutant type)	ASP	LEU	PRO	PHE	ASN	LYS	SER	ASN	LYS	ALA	ARG	SER	ARG	TYR	HIS

**Table 2:** Docking scores (in kcal/mol) and binding features for best five Cmds against Omicron BA.1.

Molecule	2D-Chemical Structure	Docking Score (Kcal/mol)	Binding Features (Hydrogen Bond Length in Å)
1	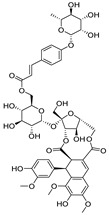	−9.7	ASP405 (2.28 Å), ARG408 (2.80 Å), TYR453 (2.86, 3.13 Å), ARG498 (3.31 Å), TYR501 (3.10 Å)
2	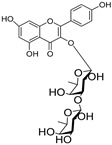	−9.5	LYS403 (2.99 Å), ASP406 (2.02 Å), GLN409 (3.08 Å), TYR453 (3.06 Å), SER494 (3.22 Å)
3	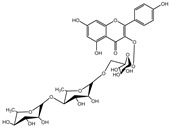	−9.2	LYS403 (3.22 Å), GLN409 (2.83 Å), VAL417 (3.27 Å)
4	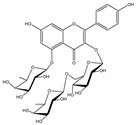	−9.0	ASP406 (2.13 Å), ARG408 (2.92 Å), GLN409 (2.90 Å), TYR449 (2.45 Å), SER496 (3.01 Å), ARG498 (3.09 Å), TYR501 (2.75, 3.13 Å)
5	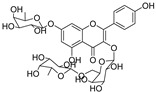	−9.0	TYR453 (2.28, 2.80, 2.95 Å), TYR449 (2.14 Å), TYR501 (2.96 Å)

**Table 3:** Toxicity risks of the five studied Cmds.

Toxicity Risk	Mu	Tu	Ir	Re
Cmd-1	G	G	G	R
Cmd-2	G	G	G	G
Cmd-3	G	R	G	G
Cmd-4	G	G	G	Y
Cmd-5	R	G	G	G

## Data Availability

Not applicable.

## Supplementary Materials

The following supporting information can be downloaded at: https://www.mdpi.com/article/10.3390/cimb44100342/s1, Figure S1: The ACE2 receptor (in blue) complexed with the O−RBD strain BA.1 (in red); Table S1: Compounds’ name and docking scores of the fifteen; Table S2: Chemical structure of the isolated compounds (1-15).
