# Supplementary material for: Targeting Natural Plant Metabolites for Hunting SARS-CoV-2 Omicron BA.1 Variant Inhibitors: Extraction, Molecular Docking, Molecular Dynamics, and Physicochemical Properties Study
Source: Curr Issues Mol Biol. 2022 Oct 19;44(10):5028–47. doi: 10.3390/cimb44100342 (PMC9600405; doi:10.3390/cimb44100342)
Supplement: Supplementary file 1 [file cimb-44-00342-s001.zip › cimb-1903255-supplementary.pdf]

Targeting natural plant metabolites for Hunting SARS-CoV-2 Omicron BA.1 Variant Inhibitors: Extraction, Molecular docking, Molecular dynamics, and Physicochemical Properties Study

Table S1 Compounds' name and docking scores of the fifteen studied compounds.

Cpd.NO	Compound Name	Docking Score (in kcal/mol)
1	Echiumin A	−9.7
2	kaempferol 3−O−(3"−O−α−L−rhamnopyranosyl)−α−L−rhamnopyranoside	−9.5
3	kaempferol 3−O−[α−−rhamnopyranosyl−(1→4)−O−α−rhamnopyranosyl−(1→6)−O]−β−galactopyranoside (kaempferol 3−O−β−isorhamninoside)	−9.2
4	kaempferol 3−O−α−rhamnopyranosyl−(1→6)−β− glucopyranosyl− 5−O−α−rhamnopyranoside	−9.0
5	kaempferol 3−O−α−rhamnopyranosyl−(1→6)−β− glucopyranosyl− 7−O−α−rhamnopyranoside	−9.0
6	kaempferol 3−O−α−L−rhamnopyranosyl (1→6)−β−D−galactopyranoside−7−O−α−L−rhamnopyranoside	−8.9
7	kaempferol 3−O−α−¬[(6−P−coumaroyl galactopyranosyl−O−β−(1→4)−O−α−rhamnopyranosyl−(1→4)]−O−α−rhamnopyranoside	−8.9
8	Kaempferol 7−O−α−L−rhamnopyranoside	−8.8
9	Kaempferol 3−O− α−l−arabinopyranosyl−5− O−α−L−rhamnopyranoside	−8.8
10	Kaempferol 3−O− rutinoside	−8.7
11	Kaempferol −3−O−α−L−galactopyranosyl 7−O−α−L−rhamnopyranoside	−8.7
12	Kaempferol −3−O−α−L−rhamnopyranosyl (1→6) (3´´−acetyl)− β−D−galactopyranoside	−8.6
13	Diferuloyl−spermidine	−8.6
14	Kaempferol 3−O− α−l−arabinopyranosyl− 7− O−α−L−rhamnopyranoside	−8.5
15	Coumaroyl−feruloyl spermidine	−8.5

Table S2 Chemical structure of the isolated compounds (1-15)

1	Echiumin A	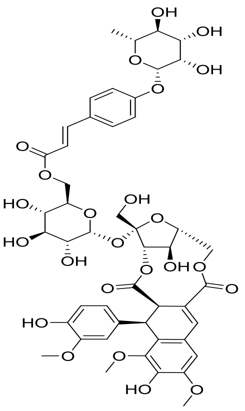
2	kaempferol 3−O−(3"−O−α−L−rhamnopyranosyl)−α−L−rhamnopyranoside	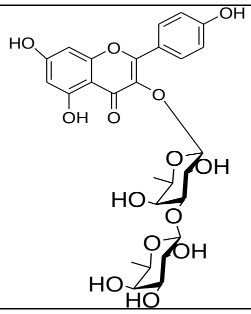
3	Kaempferol 3−O−[α−−rhamnopyranosyl−(1→4)−O−α−rhamnopyranosyl−(1→6)−O]−β−galactopyranoside (kaempferol 3−O−β−isorhamninoside)	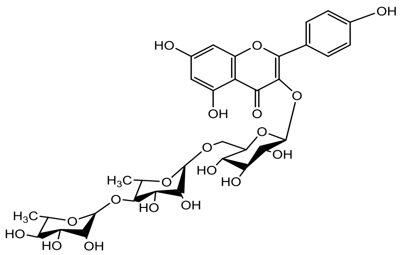
4	kaempferol 3−O−α−rhamnopyranosyl−(1→6)−β−glucopyranosyl− 5−O−α−rhamnopyranoside	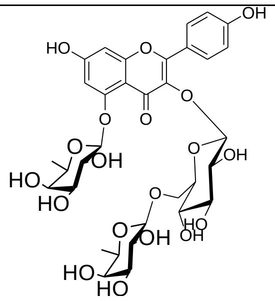
5	kaempferol 3−O−α−rhamnopyranosyl−(1→6)−β−glucopyranosyl− 7−O−α−rhamnopyranoside	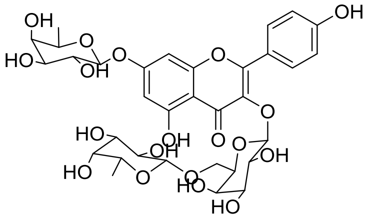

6	kaempferol 3−O−α−L−rhamnopyranosyl (1→6)−β−D−galactopyranoside−7−O−α−L−rhamnopyranoside	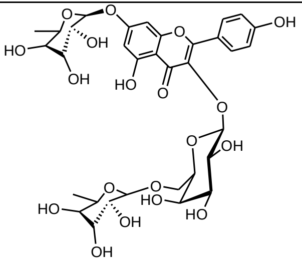
7	kaempferol 3−O−α−¬[(6−P−coumaroyl galactopyranosyl−O−β−(1→4)−O−α−rhamnopyranosyl−(1→4)]−O−α−rhamnopyranoside	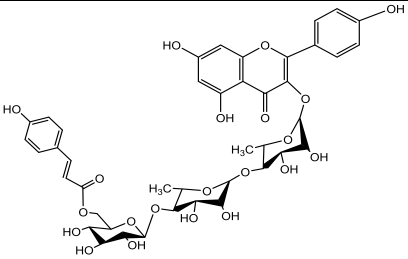
8	Kaempferol 7−O−α−L−rhamnopyranoside	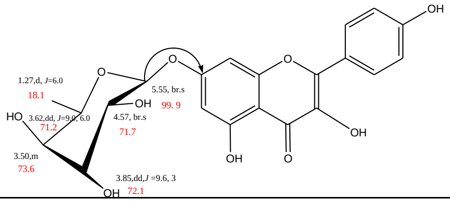
9	Kaempferol 3−O− α−l−arabinopyranosyl−5−O−α−L−rhamnopyranoside	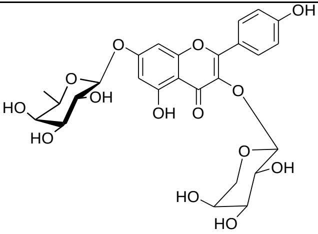
10	Kaempferol 3−O− rutinoside	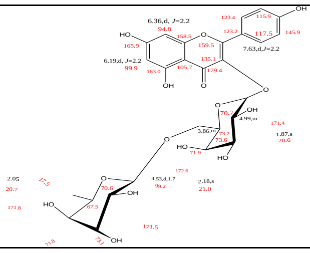
11	Kaempferol −3−O−α−L−galactopyranosyl 7−O−α−L−rhamnopyranoside	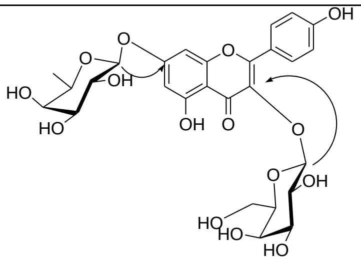
12	Kaempferol −3−O−α−L−rhamnopyranosyl (1→6) (3′′−acetyl)− β−D−galactopyranoside	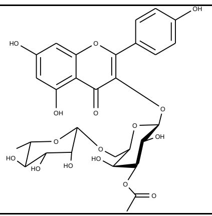

13	Diferuloyl−spermidine	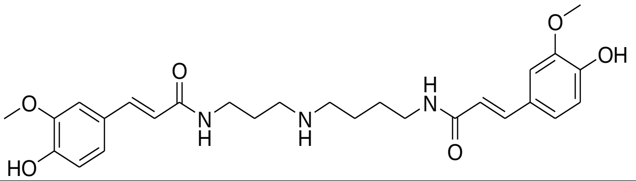
14	Kaempferol 3−O− α−l−arabinopyranosyl− 7−O−α−L−rhamnopyranoside	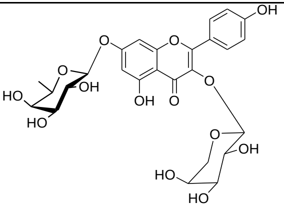
15	Coumaroyl−feruloyl spermidine	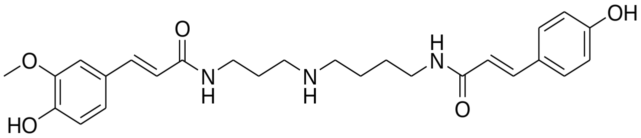

Severe acute respiratory syndrome coronavirus 2 (SARS−CoV−2) is the causative agent of the current coronavirus disease 2019 (COVID−19) pandemic. The receptor−binding domain (RBD) of the spike protein (S−protein) of SARS−CoV−2 interacts with the human angiotensin−converting enzyme 2 (ACE2) receptor and induces the viral infection. The RBD−ACE2 binding facilitates SARS−CoV−2 entry and initiates the infection process. In recent times, a number of SARS−CoV−2 mutations have been observed, and the mutations that show high infectivity are recognized as variants of concern by the United States Centers for Disease Control (US CDC). As compared to the original strain of Wuhan, the Omicron variant (OV) developed fifty genetic mutations, out of which fifteen mutations are in the RBD of S−protein. As reported in the previous study, G496S, Q498R, N501Y, and Y505H are the main mutant residues that led to an increase in the overall binding profile of the RBD−ACE2 system. Various computational approaches will be used in the current investigation to suppress mutant residues in the Omicron RBD (O−RBD), preventing it from interacting with the human ACE2 receptor.

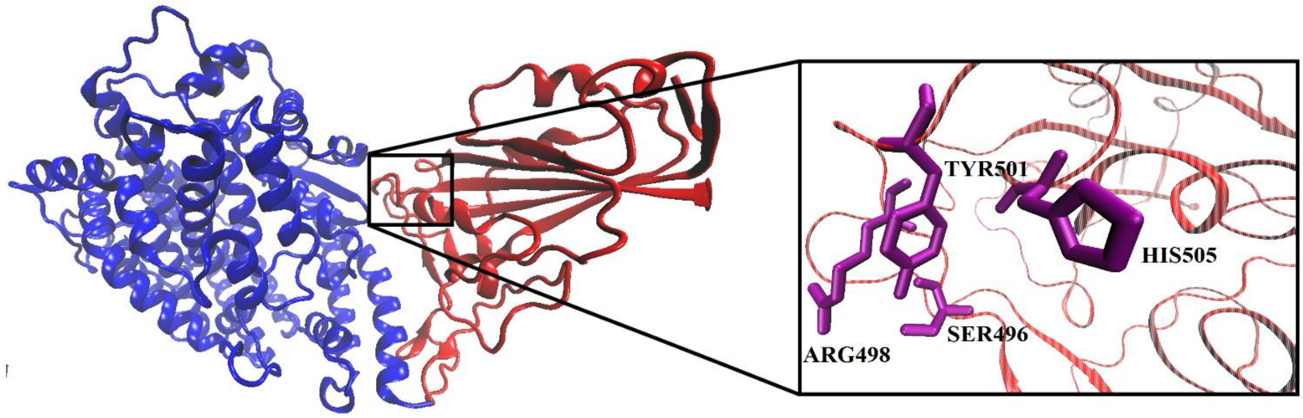

Figure S1. The ACE2 receptor (in blue) complexed with the O−RBD strain BA.1 (in red).
